# Size reduction of gastric fundic gland polyposis by de‐escalation of acid‐suppressive therapy

**DOI:** 10.1002/deo2.135

**Published:** 2022-06-05

**Authors:** Akira Kanamori, Keiichi Tominaga, Hironori Masuyama, Mutsumi Ishikawa, Satoshi Masuyama, Masayuki Kondo, Mimari Kanazawa, Takanao Tanaka, Masamichi Yamaura, Keiichiro Abe, Shoko Watanabe, Akira Yamamiya, Yoko Abe, Kenichi Goda, Atsushi Irisawa

**Affiliations:** ^1^ Department of Gastroenterology Dokkyo Medical University School of Medicine Tochigi Japan; ^2^ Masuyama Gastrointestinal Clinic Tochigi Japan

**Keywords:** acid‐suppressive therapy, de‐escalation, esophagogastroduodenoscopy, fundic gland polyposis

## Abstract

The patient, a 73‐year‐old woman, had been taking acid‐suppressive therapy for refractory reflux esophagitis for 10 years. A potassium‐competitive acid blocker was administered to strengthen acid‐suppressive therapy for worsening symptoms of gastroesophageal reflux disease. Esophagogastroduodenoscopy showed an increase in the number and size of fundic gland polyposis (FGPs). When acid‐suppressive therapy was changed from potassium‐competitive acid blocker to proton pump inhibitor, the FGPs showed reduced size 1 year later. Furthermore, when acid‐suppressive therapy was changed from proton pump inhibitor to histamine‐2 receptor antagonist, FGPs were even smaller after 1 and 2 years. The patient, who had no flare‐up of gastroesophageal reflux disease symptoms, continues to be treated medically with histamine‐2 receptor antagonist. This case report describes changes in endoscopic findings of a patient with FGPs caused by acid‐suppressive therapy for refractory gastroesophageal reflux disease.

## INTRODUCTION

Fundic gland polyps (FGP) are present in non‐atrophic mucosa without *Helicobacter pylori* infection or active gastritis. Reportedly, 0.8%‐23% of FGP have been observed using esophagogastroduodenoscopy (EGD).^1^ The polyps occur as three distinct clinical manifestations: sporadic polyp, acid‐suppressive therapy‐associated polyp, and syndromic polyp (i.e., from familial adenomatous polyposis syndrome). A large retrospective study and meta‐analysis have shown that long‐term acid‐suppressive therapy using proton pump inhibitors (PPIs) is associated significantly with FGP prevalence and incidence.^2^ Vonoprazan, of a new class of acid suppressants known as potassium competitive acid blockers (P‐CABs), has become available. Reportedly, it inhibits gastric acid secretion more strongly than PPIs do. Although the relation between FGP occurrence and P‐CAB use has not been established, the possibility exists that long‐term use of P‐CABs might be similarly involved in FGP development.

This case report describes changes in endoscopic findings of a patient with fundic gland polyposis (FGPs), presumably caused by acid‐suppressive therapy for refractory gastroesophageal reflux disease (GERD).

## CASE REPORT

**FIGURE 1:**
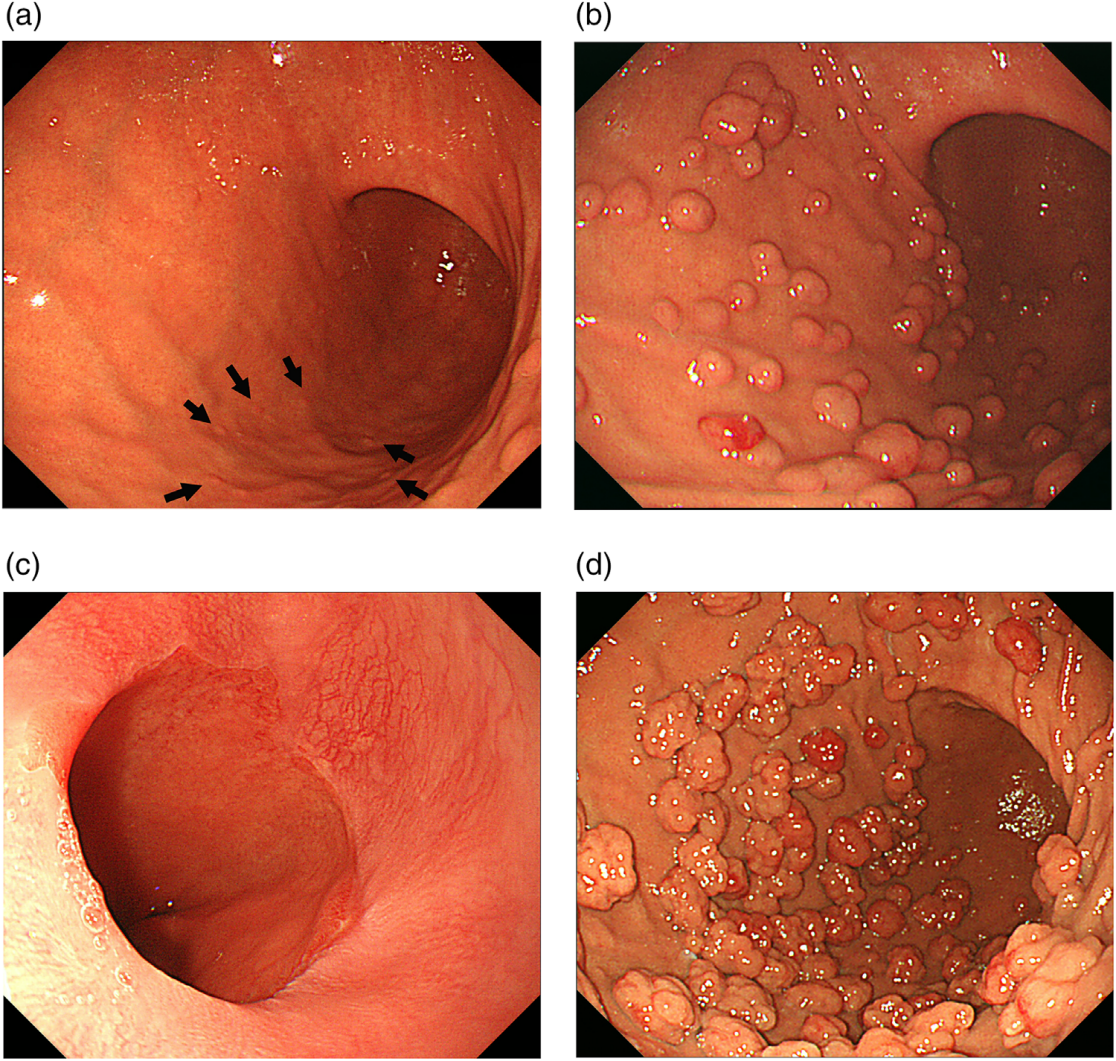
Endoscopic findings at the escalating phase of acid‐suppressive therapy. (a) Before acid‐suppressive therapy, several fundic gland polyps were found. (arrows). (b) After acid‐suppressive therapy administrated by a proton pump inhibitor, fundic gland polyps were enlarged and increased. (Lansoprazole; 15 mg/day for 10 years). (c) Residual erosions at the esophagogastric junction and Los Angeles classification Grade A reflux esophagitis. (Lansoprazole; 15 mg/day for 10 years). (d) After acid‐suppressive therapy administrated by potassium‐competitive acid blocker, fundic gland polyps were enlarged and increased further. (Vonoprazan; 10 mg/day for 2 years)

**FIGURE 2:**
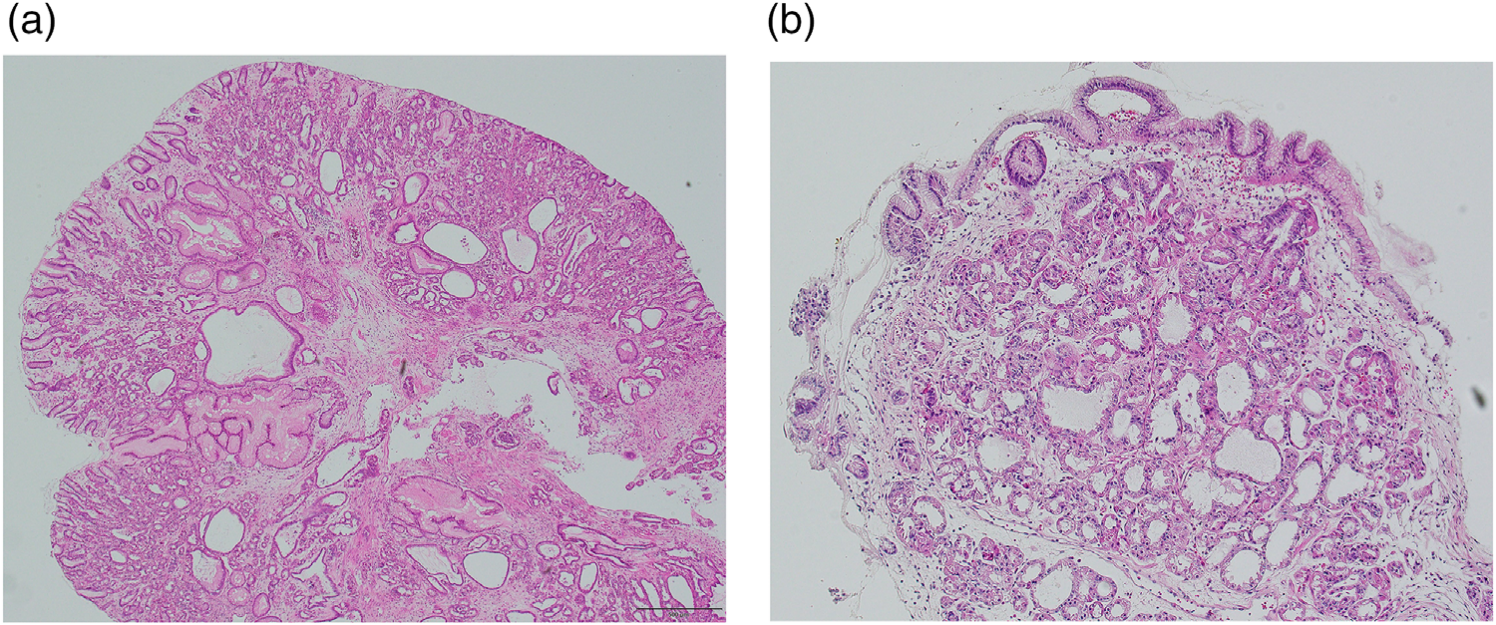
(a) Biopsy specimen obtained from the fundic gland polyp showed a typical fundic gland polyp with microcysts lined by fundic epithelium. Hematoxylin and eosin staining, original magnification, ×40. (b) Histopathologically, there were no significant changes after the de‐escalating phase of acid‐suppressive therapy. Hematoxylin and eosin staining, original magnification, ×40

**FIGURE 3:**
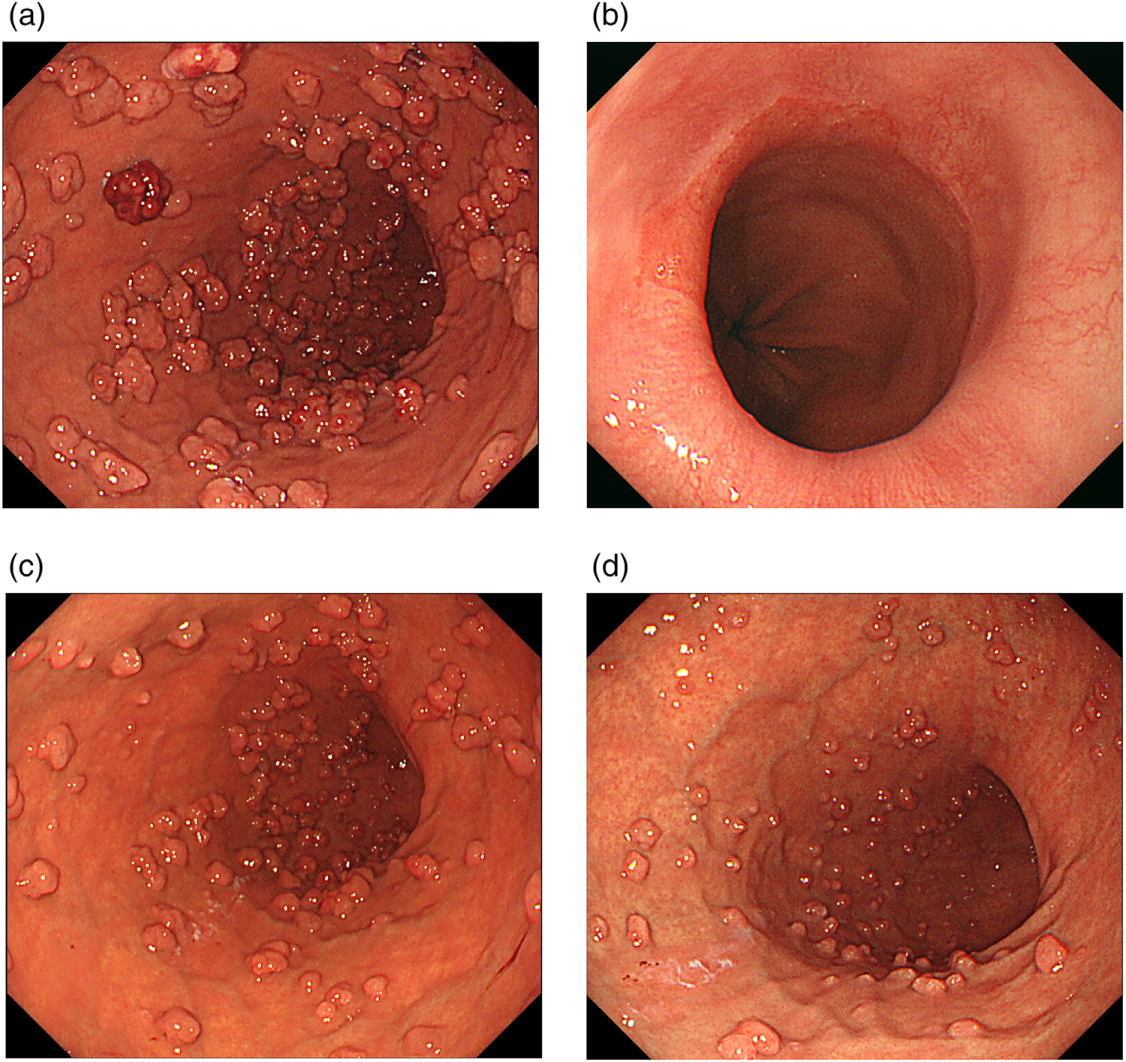
Endoscopic findings at the de‐escalating phase of acid‐suppressive therapy. (a) Size reduction of fundic gland polyposis, 1 year after the change from potassium‐competitive acid blocker to proton pump inhibitor. (b) Findings of reflux esophagitis improved. (1 year after the change from potassium‐competitive acid blocker to proton pump inhibitor). (c, d) Size reduction of fundic gland polyposis 1 year (c) and two years (d) after the change from proton pump inhibitor to histamine‐2 receptor antagonist

The patient, a 73‐year‐old woman, had been taking acid‐suppressive therapy (Lansoprazole, 15 mg/day) for refractory reflux esophagitis for 10 years. Compared with data obtained before the start of PPI administration, EGD findings showed marked enlargement and increase of FGP in the corpus of the stomach, suggesting polyposis (Figure 1a,b). A blood test showed no abnormal values. A serum anti‐*H. pylori* IgG antibody test yielded negative results (<3 U/ml: E Plate ‘'Eiken'’ *H. pylori* antibody; Eiken Chemical, Tokyo, Japan). Furthermore, the EGD showed no evidence of atrophic gastritis. Therefore, we diagnosed an uninfected stomach with *H. pylori*. The patient showed residual erosion at the esophagogastric junction and Los Angeles classification Grade A reflux esophagitis (Figure 1c). Colonoscopy showed no evidence of familial adenomatous polyposis syndrome. The patient had no family history of familial adenomatous polyposis. Subsequently, the patient had been administered only P‐CAB (Vonoprazan, 10 mg/day) for two years because of worsening refractory symptoms of GERD, such as heartburn and acid reflux (The frequency scale for the GERD [FSSG] symptoms was 15 points.).^3^ The FGPs were larger than they were during the previous EGD (Figure 1d). A biopsy specimen obtained from a polyp indicated a typical FGP with microcysts lined by fundic epithelium (Figure 2a). Although the patient's GERD symptoms improved (FSSG score was 3 points) by P‐CAB administration, she was concerned about the excessive number and increasing size of the polyps. We suspected that the FGPs had been exacerbated by the P‐CAB. We consequently switched to PPI (Lansoprazole, 15 mg/day) to change acid‐suppressive therapy. One year later, the FGP polyps were smaller (Figure 3a). Histopathologically, there were no significant changes (Figure 2b). Additionally, her GERD symptoms were stable (FSSG score was 3 points). Endoscopic evaluation of esophagogastric junction mucosa showed that no mucosal break occurred (Figure 3a). However, she remained concerned about the polyps. Therefore, acid‐suppressive therapy was changed to histamine‐2 receptor antagonist (H2RA; Famotidine, 40 mg/day). Further reductions in the FGPs size were observed after one and two years (Figure 3c,d). The GERD symptoms did not recur, even with continued H2RA (FSSG score of 3). The patient continued H2RA administration.

## DISCUSSION

Since their introduction in the late 1980s, PPIs have been used worldwide to treat acid‐related disorders such as GERD and peptic ulcers. They have also been administered prophylactically to prevent gastroduodenal mucosal damage caused by NSAIDs. The high efficacy and low toxicity of PPIs, combined with a high prevalence of GERD and NSAIDs use, have caused these to become commonly prescribed agents. GERD is a chronic condition. The most affected patients experience a symptomatic relapse if PPI therapy is discontinued. Therefore, many GERD patients require continuous maintenance of PPI therapy. An important issue with long‐term PPI usage is the increase in the level of the peptide hormone gastrin as a result of the homoeostatic response of antral G cells to the reduced acidity of gastric juice. Gastrin exerts trophic effects on the entire gastrointestinal tract tissue, including both parietal and enterochromaffin‐like cells distributed throughout the oxyntic mucosa.^4^ Long‐term use of PPIs causes histopathological changes such as protrusion of gastric wall cells into the gland lumen and cystic dilatation of the gastric fundic gland, resulting in the development of FGP. Because P‐CAB has a high pKa value and because it is stable in an acidic environment, it accumulates in the acidic compartments of gastric parietal cells. In addition, P‐CAB requires no acid activation, in contrast to PPIs. Therefore, P‐CAB can achieve potent and sustained inhibition of gastric acid secretion.^5^ However, P‐CAB is thought to induce high concentrations of serum gastrin and ultimately increase FGP as a result of the response of antral G cells, in conjunction with its potent inhibitory effect on gastric acid secretion. On the other hand, de‐escalation of acid‐suppressive therapy is speculated to increase the acidity of gastric juice and improve hypergastrinemia, resulting in a reduction of FGP. In this case, the endoscopic findings changed according to that speculation.

The most commonly reported gastric polyp is FGP: benign lesions that might be sporadic or caused by an underlying genetic mutation. Endoscopically, FGP is a sessile polyp located in the gastric corpus and fundus. Their surface color is indistinguishable from the surface of the normal gastric mucosa. Most endoscopists are reportedly able to diagnose FGP with 89% accuracy by endoscopic findings alone.^1^ Microscopically, FGP comprises the dilated glands covered in gastric mucosa without malignant change. Each lesion is 1–8 mm in size. FGP occurs more commonly in middle‐aged women. Colonoscopy of the patient showed no evidence of familial adenomatous polyposis syndrome. She had no family history of familial adenomatous polyposis. Therefore, the FGPs were regarded as associated with acid‐suppressive therapy that was increased by long‐term acid suppression of GERD.

In this patient, FGPs size reduction occurred over time as the acid‐suppressive therapy strength was changed from P‐CAB to PPI to H2RA. Actually, FGP is characterized by a spontaneous decrease or increase in the number of polyps. Usually, FGP resolves without symptoms, but rare complications have been reported. Behrens et al.^6^ reported the case of a 70‐year‐old woman who developed gastric intussusception secondary to FGP. The patient discontinued PPIs. No recurrence of symptoms occurred. Although the risk of cancer development from FGP is very low, rare cases of neoplastic lesions such as adenoma, carcinoma and foveolar‐type gastric carcinoma have been reported.^7^, ^8^ Furthermore, de‐escalation should be considered for unnecessary acid suppression because of a case report of gastroduodenal intussusception with FGPs caused by P‐CAB administration.^9^ In addition, Japanese evidence‐based clinical practice guidelines for gastroesophageal reflux disease 2021 state that “when treating GERD with PPIs, their dosage and administration must be as low and short, respectively, as possible”.^10^ Although acid‐suppressive therapy has no major adverse event, a slight risk of intestinal infections, bone fractures, and microscopic colitis has been reported with long‐term use.^10^ Therefore, de‐escalation of acid‐suppressive therapy is recommended not only for FGPs prevention but also for GERD patients whose symptoms have improved. However, in patients with refractory GERD who are unable to reduce the dosage of acid‐suppressive therapy, including P‐CAB and PPIs, the guidelines call for the use of a gastrointestinal prokinetic agent such as mosapride or baclofen after optimizing acid‐suppressive therapy.^10^ Annual endoscopic surveillance is important to optimize acid‐suppressive therapy and to detect FGPs in patients with severe GERD for whom acid‐suppressive therapy, including P‐CAB, cannot be changed. The development of neuroendocrine tumors (NETs) associated with hypergastrinemia should be considered independently. The NET is a subepithelial tumor and should be carefully observed at the time of surveillance endoscopy.

The prevalence of FGP is expected to increase along with the widespread and frequent use of PPIs and the decreasing infection rate of H. pylori. Currently, it remains unclear whether carcinomas caused by FGP will also increase similarly to FGPs. Therefore, until evidence has settled these points, we recommend that regular endoscopic surveillance be performed in cases requiring acid‐suppressive therapy. The study of this valuable case using long‐term observations by EGD has revealed aspects of FGPs size reduction that occurs with the de‐escalation of acid‐suppressive therapy.

## CONFLICT OF INTEREST

The authors declare that they have no conflict of interest.

## FUNDING INFORMATION

None.

## ETHICS STATEMENT

This case report was conducted in accordance with the ethical standards laid down in the 1964 Declaration of Helsinki and its later amendments.
